# Sexual and gender diversity in a Métis community: challenging stigma and celebrating resilience

**DOI:** 10.1177/11771801251334914

**Published:** 2025-04-28

**Authors:** Allison Reeves, Rachel Landy, Shining Mountains Living Community Services

**Affiliations:** 1 University of Guelph-Humber, Canada; 2 Department of Psychology, University of Guelph, Canada; 3 School of Health and Human Performance, Dalhousie University, Canada; 4 Métis Community Charity, Canada

**Keywords:** gender diversity, Indigenous community-based research, Indigenous healing, Métis mental health, sexual diversity, stigma

## Abstract

Shining Mountains Living Community Services in Red Deer, Alberta, Canada, provides a range of social services for Indigenous Peoples in the area, particularly Métis (an Indigenous people of Canada), who are struggling with sexual health vulnerabilities. Under the direction of a Métis Wellness Advisory Council at Shining Mountains, this study sought to understand how stigma and discrimination affect sexually diverse and gender-diverse Métis community members. Grounded in qualitative interviews and Métis methods including sharing circles and visual arts, this study looked at experiences of stigma, resilience, and healing for Key Informants including sexual or gender-diverse Métis Peoples. This article details findings from eight Key Informant interviews and discusses major themes related to Layers of Stigma, Métis Identity and Teachings, and Resilience and Healing. Conclusions offer directions for mental health service development and community healing.

## Introduction

Shining Mountains Living Community Services (Shining Mountains) in Red Deer, Alberta, Canada, provides a range of social services for Indigenous Peoples, particularly Métis (an Indigenous people of Canada). Shining Mountains is an organization that celebrates sexual and gender diversity, consistent with what is known of many pre-colonial Indigenous cultures in the region who demonstrated respectful and more open attitudes toward sexual and gender diversity (Reeves & Loppie, 2012). Unfortunately, colonial impacts, such as Euro-Christian patriarchy and intergenerational sexualized trauma from residential schooling, have undermined some of these traditional values related to positive sexuality and acceptance of diversity (Reeves & Loppie, 2012). As a result, many Indigenous Peoples today are disproportionately impacted by higher rates of sexual vulnerabilities, including sexually transmitted infections, sexualized violence, and stigma related to sexuality and gender identity (Andersson et al., 2008; Majumdar et al., 2004; Reeves & Stewart, 2017). Currently, Shining Mountains supports Métis Peoples, and other Indigenous Peoples in the area, who are living with, or who are at risk of, HIV/AIDS and other STIs, homelessness, domestic violence, and addictions.

In 2020, Shining Mountains struck a Métis Wellness Advisory Council (MWAC) in response to community challenges facing those experiencing mental health vulnerabilities related to sexual and gender minority identities (2SLGBTQ+). Métis Wellness Advisory Council then partnered with our research team to look specifically at gender and sexuality-related stigma and its reverberating impacts on mental health in the local Métis community. Over the course of 2 years, the team undertook a community-driven research study, inclusive of Métis cultural practices, to consider the following research questions: *(1) What are Métis cultural and community-based models and understandings of stigma and resilience, as they relate to sexual and gender vulnerabilities? (2) How can these understandings inform community programs to reduce stigma directed at sexually diverse and gender-diverse Métis community members?*

## Context of Métis health, sexual vulnerabilities, and stigma

Métis Peoples represent a distinct Indigenous group in Canada and are a people of mixed Indigenous and European ancestry with a shared culture and history dating back to the first waves of European colonialism (Adams et al., 2013; Macdougall, 2017). Métis homelands tend to connect back to the Métis Nation of the prairie provinces, though the wider Métis territory extends into other parts of Canada and into the United States (Adams et al., 2013; Sprague, 2009). Métis identity has been challenged by many, including by other Indigenous groups, for being too White to be Indigenous, and therefore inauthentic in its Indigeneity; Métis Peoples have at times considered themselves the *forgotten people* and rights to Indigenous status in Canada have been hard-fought by Métis communities over generations (Macdougall, 2017). Despite these questions of identity, Métis Peoples, like other Indigenous groups in Canada, were also dispossessed from traditional lands by the new colonial governments, altering their traditional land-based economies, interrupting community structure, and leading to social and economic marginalization. As with other Indigenous Peoples, some Métis Peoples also experienced the forced removal of their children into residential schooling, as well as the overrepresentation of Métis children in the child welfare system (Gmitroski et al., 2023), among other colonial injuries.

Health inequities among all Indigenous Peoples in Canada have links to colonial harms related to denying cultural rights and language, land dispossession, economic marginalization, and forced attendance in residential schooling, with its subsequent disruption to family and community structure (Reeves & Loppie, 2012; Reeves & Stewart, 2017). Present-day sexual health inequities and vulnerabilities must likewise be considered through an understanding of colonization and social inequities. Colonization brought with it Euro-Christian values of patriarchy, male dominance and female passivity, sexuality-as-sin, as well as sexual norms that often scripted Indigenous peoples as sexually corrupt (Carter, 1997). Colonial practices of Christianity often sought to issue social control through teachings of *original sin*, for instance (Million, 2000), and gender diversity beyond the binary was unacceptable within this religious framework (Wolff et al., 2017). Through the residential schooling system, students endured years of physical, emotional, and sexual abuse, leading to what is now understood in the psychological literature as *complex trauma* (Haskell & Randall, 2009; Herman, 1992). Intergenerational trauma and sexual vulnerabilities for Indigenous Peoples have since been linked to cycles of violence from residential schooling and the foster system, systemic racism and chronic stress, economic and social deprivation, alcohol and substance abuse, and the overall impact of colonization on traditional values and cultures (Reeves & Loppie, 2012; Reeves & Stewart, 2015, 2017).

Today, Indigenous Peoples in Canada, and particularly youth, are disproportionately impacted by sexual vulnerabilities, including sexual stigma related to sexual and gender identity, sexually transmitted infections and blood borne illnesses, and higher rates of sexualized violence (Andersson et al., 2008; Majumdar et al., 2004; Reeves & Stewart, 2017). Health outcomes are directly linked to social inequities experienced by Indigenous Peoples, as explained here, and are impacted by a series of factors including poor access to care due to geographical barriers, poor access to testing, and a lack of follow-up care, among others (Negin et al., 2015; Skinner et al., 2018; Wynne & Currie, 2011). Importantly, encounters with covert and overt racism are often ubiquitous in the lived experience of Indigenous Peoples in Canada; in this sense, racism also represents a critical social determinant of health for Indigenous Peoples (Bourassa et al., 2005; Nelson & Wilson, 2017). A recent scoping review in Canada by Cooke and Shields (2024) found that racism negatively impact Indigenous patient experiences within mainstream healthcare systems, leading to a reluctance to seek out care and to higher unmet healthcare needs. In the context of addressing sexual vulnerabilities, racism is a further barrier to obtaining culturally safe, timely, and appropriate care.

Experiences of social stigma and resultant shame feature prominently among those who experience sexual and gender vulnerabilities (Reeves & Stewart, 2015, 2016). Within social environments, sexual norms and gender scripts set a tone of what is right or wrong, and moral or amoral; sensing that one is on the *wrong* side of these dualities can lead to anxiety and internalized shame (Foster & Byers, 2008). For instance, individuals may cast their own identity in a negative light for defying standards of heteronormativity, what it means to be a male or female, or for being sexually *promiscuous* (Pescosolido, 2013; Wynne & Currie, 2011). Taken together, scholars involved in Indigenous and queer activism have described colonial violence through an intersectional lens, considering racism against Indigenous peoples as well as violence fueled by homophobia (Holmes et al., 2015), identifying that dehumanizing colonial norms harm both Indigenous and queer community members—and particularly queer Indigenous peoples—through stigma.

Stigma and shame experienced by Indigenous Peoples represent an ongoing threat to their overall well-being and pose particular challenges when associated with sexual and gender vulnerabilities (Reeves & Loppie, 2012; Reeves & Stewart, 2017). Stigma relates to experiences of ostracism, marginalization, and social devaluation (Audet et al., 2013; Pescosolido, 2013). Through social shame, individuals self-isolate, engage in avoidance coping strategies, and often refrain from asking others for help (Omorodion et al., 2007; Reeves & Stewart, 2017). For youth specifically, it is difficult to receive transparent sexual health education and support around gender and sexual diversity if youth do not feel safe speaking about these topics with peers, adults, and care providers (Askew, 2007; Lys & Reading, 2012; Reeves & Loppie, 2012).

## Study objectives

The central aim of this study was to explore Métis youth, adult, and Elder experiences around sexuality-related and gender-related stigma, as well as to capture culture-based teachings from youth and Elders related to coping and resilience for positive sexuality and gender identity. This study brought together youth, adults, and Elders in the Red Deer Métis community, primarily through affiliation with Shining Mountains, to meet the following research objectives: (1) *Knowledge Creation*: This study sought to contribute to cultural resurgence efforts in the creation of Métis cultural and community-based anti-stigma models and understandings around positive sexuality and gender for community youth, grounded in an intergenerational healing model that is rooted in the knowledge and lived experience of community Elders, adults and youth; and (2) *Enhanced Community Research Capacity*: This study sought to build capacity through the promotion of community and academic engagement in culturally safe research, through the enhancement of research skills among youth and other stakeholders, and through the creation and utilization of culturally relevant arts-based research methodologies.

## Methodology

### Background: building on existing successful research

This project emerged from the extensive work of the Development of a Rural Model (DRUM) project for integrated shared care in First Nation and Métis Communities in Northern Alberta, and the DRUM & SASH—referring to the sash, which is symbolic in Métis culture—project, renamed to include the Métis partnership. These research projects, funded over an 8-year period, partnered with First Nation and Métis communities in Alberta around sexually transmitted infection prevention and care services. The large interdisciplinary DRUM & SASH team was composed of local Elders and community members, community health workers, community research coordinators, people with lived experience, and academics representing a range of disciplines, including members of this Stigma Study research team.

What emerged through the DRUM & SASH collaboration with Métis partners was a specific need to consider stigma, coping and resilience for sexually diverse and gender-diverse people who experience discrimination. The MWAC driving the current research project sought to address stigma and promote healing through this study, using a lens that considered cultural mental health promotion.

## Methods

The research team was composed of Métis community health workers and advocates at Shining Mountains, the MWAC team comprised of three Métis youth, two adults, and four Elders, and both Indigenous and non-Indigenous academics with expertise in Indigenous mental health and cultural resurgence, sexuality and gender studies, and culturally safe research methods. The research methods involved qualitative narrative and arts-based methods that were culturally relevant and consistent with Métis oral and expressive traditions, including gathering circles, storytelling, qualitative interviews, and culture- and arts-based data collection methods (Landy et al., 2022; Lys et al., 2016; Reeves & Stewart, 2017). These arts-based approaches are detailed in a forthcoming paper examining Métis-informed health research methods. The findings in this article focus on results from qualitative interviews and sharing circles, uncovered using community-based qualitative analysis methods.

**Figure 1.**
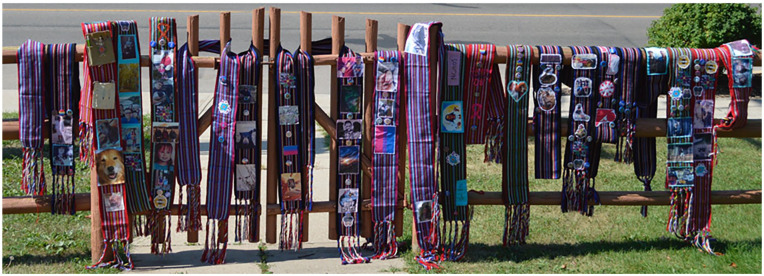
Story telling through the Métis sash (photo by Allison Reeves). Métis = an Indigenous people of Canada.

With ethical approval from the University of Guelph and the University of Victoria, the first community gathering took place in August of 2022 in Red Deer, Alberta, Canada. Community participants included both those with lived experience as a gender-diverse or sexually diverse person henceforth referred to as Key Informants as well as Métis allies, who are not gender-diverse or sexually diverse. Participants were recruited through word-of-mouth through MWAC connections and through posters at Shining Mountains. The participants utilized traditional Métis sashes to tell the story of their lives, including experiences of stigma and discrimination, as well as healing, using a photovoice method (Figure 1). Individual interviews also took place, which asked participants about experiences of stigma and resilience. The second gathering took place in Red Deer in December of 2022 and the academic research team met with the MWAC council to engage in participatory qualitative analysis. This process loosely followed the DEPICT (Dynnamic reading . . . Engaged codebook development . . . Participatory coding . . . Inclusive reviewing and summarizing of categories . . . Collaborative analyzing) model developed by Flicker and Nixon (2015, p. 618) to engage community members in developing thematic codes from interview transcripts, which the authors describe as a collaborative approach to qualitative data analysis. In our case, this involved pairing a research team member with a Métis community member, and, using a low-tech method with printed transcripts and highlighters, following a dynamic reading of the transcripts to identify possible codes, drawing from combined lay, professional, and academic experience. This was an iterative process that involved reading and re-reading transcripts, engaging in participatory coding, reviewing and summarizing categories as a larger group, and developing a final codebook as a team. This engagement allowed for community members themselves to translate the meanings held in the interview data in the development of final themes. Following this second community gathering, the academic research team met to review transcripts using the master codebook and created a summary of major topics, themes, and findings that emerged from the community-based analysis gathering.

## Results

It was noted in the Methods section that data collection involved interviews with Key Informants. This article will detail the findings from the Key Informant group; the results sharing the stories and voices of allies are published in a separate paper looking at empathy, relationship, and allyship. Key Informant findings discussed here include the voices of one Elder, one adult participant, and six youth ranging in age from 14 to 30, and findings are presented in narrative form, showcasing the voices of each of the participants, in keeping with Indigenous methodologies (Kovach, 2005).

### Elder

The Elder described the difficulties he experienced as a gay man in the 1980s and 1990s, both in the community and in the workplace. He recalled fearing the loss of family and friends through coming out, and he recalled having to make the argument to others that “being gay is not a disease.” When seeking a leadership position at work he shared that others told him: “We are not ready for a person like you.” He noted that in those years, “there was no acceptance of being gay.” Likewise, he recalled experiences of stigma due to his Indigenous status: “In the 80’s, nobody wanted to be an Indian.” He later shared, “They never recognized ‘Métis.’” He noted the silence around mental health concerns also.

This Elder offered his view that things have changed considerably over the years; he noted there is now “an openness” for sexual and gender diversity and that he may have helped to “open doors for people” in this sense. He offered the example that people who once rejected him have now become his friends, years later. “Things changed,” he shared. He added that “It’s going to be the youth that are making the change” moving forward.

In terms of healing, the Elder described the importance of allyship during difficult years, noting that all along he had friends and members of his church community who respected and supported him. He acknowledged this need for social belonging and shared that he now feels: “I’m a proud Métis man. I’m also a proud Métis gay man.” He described the importance of maintaining Métis customs and traditions for mental wellness. As a fluent Cree-Michif (a Métis language, which is a blend of Cree (an Indigenous language of the Cree Nation), French, and other Indigenous languages) speaker, he shared at interview, “I am Métis. I am distinct . . . I am unique.” In terms of healing practices, he noted that he follows both “Native spirituality” as well as Christian traditions, engaging in sweat lodge ceremonies, sharing circles, as well as attending Church on Sundays. He recalled a Healer encouraging him to accept that he “was a Two-Spirited person”; this Healer offered the following resilience teaching: “If you don’t walk on the path you’re meant to walk, you will never experience happiness.” He added, “I am gay. I am human . . . if you have an issue, it’s your issue, because I can live with myself . . . Who do you think made me? God made me, just like he made you.”

### Adult

The adult participant, who was over the age of 50, identified as both a sexually diverse and a gender-diverse person and humorously referred to themselves as “Vintage” due to their age. They shared that they experienced stigma as an “every week kind of experience” in their life. They offered a range of examples, including people driving by and yelling “faggot,” being seen as a “pedophile” or a “man in a dress,” as well as experiencing homophobia from within the Métis community. They added: “I think it scares some people just by being who I am. I mean, that’s the definition of phobia, right?” They shared difficulties finding employment over the years, as “it used to be that they didn’t like to hire gay people. In fact, in the 90’s and 80’s you could still be fired if they found out you were gay.” They noted that things have changed in some respects, but not entirely. While it’s no longer legal to fire employees due to being gay, they shared, “They will find another reason to fire you.” They suggested that stigma has likely lessened for the current generation of youth and that the “Vintage” generation was unable fight all battles on behalf of today’s youth as they were, “busy trying to survive.” Still, they noted: “We feel the guilt of not teaching the younger ones enough that this could happen again,” referring to ongoing oppression.

This participant shared a sense of connection to their Métis identity by history, family, and DNA. They shared that Indigenous ways of knowing historically did not include a gender binary and a “Two-Spirit person doesn’t necessarily have to be gay.” They noted that as a Métis person, they “walk in both worlds” and that at times they feel like an outsider to normative Canadian culture due to their Indigenous identity: “We’re all trying to find our way here as Indigenous people . . . they just don’t like us at all no matter what.” They shared, “I think as Indigenous people we have all experienced stigma and discrimination at some point. . . . So we have a lot to learn from each other about when things are happening and how we deal with it.”

To cope with challenging experiences, they offered a series of wellness practices they find helpful, including taking a walk while ruminating on “expletives” they would want to say to those who have behaved in a discriminatory manner, taking a bath to relax, watching dark humor on television that normalizes difficulty, engaging in a sweat lodge ceremony, and smudging at home. They shared an experience of reclaiming a public space where they had experienced physical violence by proudly being “out” in appearance and behavior in that space. They shared, “[I went] right in that same spot where I got beat up the next week. Because it was my way of taking that spot back.”

### Youth, 28 years old

One female-identified bisexual youth shared that growing up she felt like a cultural outsider because “I was always too Native for the White people in my class . . . but the Native kids wouldn’t [hang out with] me because I was too White for them.” She found comfort in regularly attending a Métis summer camp, “where there were other people like me—it was amazing.” She shared that more recently she started to question her identity once more: “There was this person on TikTok who said, like, if you are White passing or presenting, then you’re just White . . . It took me back in a spot where I was like, ‘What am I’?” She also shared a recent experience of racial violence wherein a Métis friend wearing a ribbon skirt was attacked, possibly due to being “out” as an Indigenous person. In terms of sexual stigma, this youth shared that she experienced discrimination from a grandparent after coming out: “So it was like, ‘You’re going to hell if you do anything!’ . . . It got to the point where I just stopped talking about it with her.”

Despite these setbacks, this youth also recalled other family members responding positively to her sexually diverse identity. She recalled her mother being accepting and her step-father stating, “Well, if you’re with a girl, you can’t come home pregnant.” She also shared a touching response she received from her grandfather:

My grandpa was my hero. He was the first one I told that I liked girls and boys and he was very accepting. He told me he didn’t care who I ended up with so long as they treated me well.

She recalled there likewise being an openness of diversity at Métis camp: “We had one guy—everyone would make assumptions that he was Two Spirited, because he had a flamboyant personality. And nobody judged him on it.”

In terms of healing, this youth shared that she continues to aim to learn about, and to practice, her Métis culture. At interview, she recalled that she used to love hearing Elders share their stories. She went on to recount her favorite story of the White Buffalo Woman who helped a starving village. She shared that she could relate to the story because, “I will always give everything I have to those I care about.” She noted the importance of passing on traditions: “Our son is learning about our heritage, what has happened, and being able to share it with him helps as well.”

### Youth, 26 years old

One gender queer and gay youth expressed experiencing identity issues with respect to how to portray themselves, as they are “White passing.” They shared, “I always just felt inside, internally, I was Indigenous. But on the outside was not.” They stated, “Socially, I’m Indigenous, but not, like, racially.” They recalled that a grandparent would share Indigenous teachings but that it was “like a secret” in the family. These teachings competed with their Catholic upbringing and this youth felt confused: “I thought there was something wrong with me . . . like, I was going to hell because I believed in a Creator instead of God.” Later they received their Indigenous name, which brought “a lot of internal meaning . . . that just kind of holds a connection, like, between my family and like, my Indigenous side.” They noted they now feel settled in their Métis identity.

At interview, they shared a similar struggle when coming out as gender queer: “I have to defend myself again . . . convince others I’m human enough,” as people may think “there’s something wrong with you—something tragic must have happened to you if you’re gay.” They saw being both Métis and queer as barriers to overcome: “People [are] like, ‘Oh well, you’re pretty White’ or ‘You don’t look gay’ . . . Maybe I will respond and be like, ‘Well, that’s who I am.’” They shared in interview that they will disclose their identity on a situational basis as a result, and that they chose to sometimes hide their identity due to stigma and discrimination: “I’m stigmatized for being Métis. I’m stigmatized for being gay. At the same time, I’m that together. Two layers.”

In terms of healing, this youth shared that Indigenous teachings from Elders around diversity have been helpful, as teachers shared the importance of “embracing the masculine and feminine,” “identifying with both energies,” avoiding the gender binary, and emphasizing women in leadership roles, alongside men. They shared the example that their grandmother made them both a ribbon skirt for ceremony as well as a ribbon shirt, typically gifted to men. They shared that due to encouragement from their grandmother they were able to “blend my Métis identify and also my sexual identity, . . . that was cool to see it together . . . kind of together as Métis and queer.” They added that “being Métis kind of made me, like, already familiar with living in two worlds” and that they now feel “empowered and educated.” They noted that in any healing journey, “Just think of climbing a mountain. It’s not easy. But the top is really beautiful. So maybe after all the pain and shame and whatnot, there’s something good that comes out of it . . . and being able to accept myself.” They added that speaking with others about their difficulties has been healing, as well as practicing yoga, and spending times outdoors.

### Youth, 23 years old

One female youth identifying as bisexual shared that although she is now in a relationship with a man, she encountered earlier social difficulties when dating another woman. She shared that her best friend “completely ghosted me right after I told her” and that “I basically cut myself off from everyone” following these events because of her perception of social stigma. She noted, “It was hard. Even just admitting it to myself that I was bisexual . . . I felt dirty liking women.” She shared that her immediate family was accepting at the time and that another gay friend was a positive role model: “He kind of gave me a little bit of the confidence that I needed . . . like, This is okay. I’m allowed to do this.” She shared that she later confronted to this friend: “I told her: ‘It’s still me. Like, I haven’t changed.’” She added that she did feel stigmatized by some relatives who “gave me weird looks,” and she felt interrogated by one family member who asked her, “Why are you bisexual?” She stated at interview that she had replied: “It’s just who I am. I’m attracted to girls. Deal with it.” She stated, “For me, the impact was: you don’t accept me. If you don’t accept this big part of me then you don’t really know me.” She noted that she hadn’t realized how much this had affected her until she cried in the interview: “I never actually realized that I had a lot of hurt. Apparently, I just kind of bottled it all up and ignored it.”

This youth identified that healing for her involves sitting by herself to think, and then discussing her concerns with a close loved one. She added that when she is able to share the burden of her pain, “It feels like a weight is lifted off my shoulders.” She stated that she currently feels like “an educated, empowered Métis woman” and that adding the Métis pride flag to her sash felt meaningful as she feels as sense of belonging being “part of that community.”

### Youth, 17 years old

One male youth identifying as pan-sexual, shared that he has experienced stigma and discrimination from within the family as well as from the wider community. This youth used the term *pan-sexual* to connote they were “open to dating anyone.” He shared, “I’ve had parents say I’m not allowed near their kids . . . It’s very discriminatory . . . Just seems unfair, the stereotype . . . its extremely hurtful.” He shared that after coming out to his ardently Christian parents, their relationship fell apart, and he was “pushed out of their lives.” He added that at school he has seen students “harassing” sexual and gender minority students, and he has had to “stand up for other people” as well as “stand up for me.” At interview, he felt it is a lack of education that promotes “close-minded, negative thinking” as well as the behavior of “pushing ideologies” intergenerationally.

In addition, this youth added that he has felt stigmatized from other members of the Métis community for not knowing about Indigenous traditions, and shared that he is cautious of approaching Elders with his experiences as a sexual minority out of fear that these Elders will be “close-minded about it as well.” He shared that he hopes to be “the generation to be able to teach [acceptance of diversity] and maybe the next generation will be more educated about it.” This youth noted that dealing with discrimination due to his sexual identity helped him to better understand his Métis identity. He shared: “I’ve learned a lot about what Métis people have gone through . . . I don’t know . . . It helped me be way more open-minded.”

### Youth, 16 years old

One youth identifying as gender non-binary and bisexual shared that they feel accepted both as a Métis person and as a person of diverse gender and sexual identity. They noted, “I don’t necessarily feel excluded. Everyone’s a lot more accepting now. Because I have a lot of trans friends. They’re all accepted and supported.” They recalled one instance of a teacher using a student’s *dead name*—the name assigned at birth that the student opted to change as they transitioned gender—but otherwise did not recall instances of observed discrimination. They suggested that having close friends “who don’t judge you for anything” is key to healing from stigma. They suggested that things can improve for individuals feeling hurt by stigma: “It might not be great now, but it will get better.” In terms of their Métis identity, they noted briefly they feel it’s an “important part” of who they are and that it “shapes me as a person.”

### Youth, 16 years old

Finally, a youth who identified as asexual and non-binary in their gender identity, shared that they hope to avoid stereotypes about how a non-binary person should look: “I don’t really care what I wear, or how I look. It’s more just how I identify and what feels comfortable.” They shared that they have experienced being misgendered: “I use ‘they/them’ pronouns and some people don’t know that—which is okay—but others that do, just don’t use ‘them’ sometimes.” Otherwise, they shared that they feel “pretty accepted anywhere—honestly.” They added “I’m in a pretty nice friend group and a pretty good community that’s not discriminatory.” Although once their brother refused to use their preferred pronouns, this youth explained: “he got well yelled at by my family,” resulting in this youth feeling supported overall. This youth suggested that in order to cope with stigma, one should “Just be yourself.” They added, “Try not to let it get to you, because who cares what other people think? It’s you—you should care about yourself.” In terms of their Métis identity, they shared “I don’t know” when asked what being Métis means to them.

## Discussion

In considering the findings from Key Informant narratives, including one Elder, one adult, and six youth, the following themes emerge for discussion: Layers of Stigma, Métis Identity and Teachings, and Resilience and Healing.

### Layers of stigma

Research participants with lived experience as a sexually diverse or gender-diverse Métis person named a variety of challenges they confronted as part of their ongoing encounters with stigma. They noted that they experienced *double* discrimination: racism as a Métis person, as well as experiences of homophobia and/or transphobia, depending on their social location within the 2SLGBTQ+ umbrella. Broadly, they noted experiences of verbal slights, harassment, micro and macro aggressions, having to field stereotypes, as well as often feeling socially misunderstood and isolated. Participants shared that their experiences of sigma and discrimination were not only enacted by the non-Indigenous straight, cisgender community; they described experiencing lateral violence from members of the non-Indigenous Queer community in the form of racism due to their Métis identity, as well as lateral violence from within the Métis community in the form of homophobia and transphobic messaging.

Experiences of *double* discrimination described here align with Kimberlé Crenshaw’s (1989) theory of intersectionality, which suggests that the overlap of marginal identities—Indigenous and queer in this case—leads to a unique experience of oppression. The stories of participants in this study highlight the compounding nature of oppression and the emotional and psychological strain of racism and queer phobia on their well-being. This also aligns with work of queer and Indigenous scholars in Canada: In their article on *Everyday Decolonization*, Hunt and Holmes (2015) note the intersectionality of heteronormativity and settler colonialism as a particular pain point for queer Indigenous folk, that seeks to promote the erasure of Indigenous peoples while imposing a binary gender system that ignores traditional Indigenous teachings on sexual and gender diversity. What further complicated matters for our Key Informants was the experience of lateral violence, which is described in Indigenous literature as relating to internalized colonialism, wherein the perpetrator of lateral violence mimics the oppressor; there is an effort to control and disempower, just as that individual has experienced from the oppressor (Whyman et al., 2021). Impacts of lateral violence can include shame, feeling disconnected, poor self-esteem, and fractures within communities. While many individuals in this study understood that members of dominant culture can enact harmful discrimination, a—perhaps uncomfortable—realization was that even within their own marginalized communities they did not always experience safety. This resulted in an inability to relax or feel safe in any context, causing further stress and anxiety.

Notably, these experiences of stigma varied based on other factors, such as age/generation, and family culture and attitude. For instance, *vintage*—Elder and adult—participants described growing up with more severe experiences of discrimination and abuse, including encounters with physical violence. Some youth participants described experiences of social ostracization and “othering,” but did not name encounters with physical violence. Other youth participants shared more optimistic perspectives, stating that they experienced fewer encounters with stereotypes or interpersonal difficulty due to their sexual or gender minority status, and felt less worry and stress as a result. While we acknowledge a small sample size for this Key Informant group, which is not intended to be generalizable, it is worth noting these reports of a reduction in stigma and discrimination across generations. Although members of older generations often felt targeted, socially undesirable (Goffman, 1963) and stigmatized, which might have led to internalized shame and blame (Foster & Byers, 2008), many younger participants voiced having a strong self-concept with positive core beliefs of self.

### Métis identity and teachings

The importance of Métis identity in promoting a sense of self and positive identity construction also emerged from these interviews. Across all ages, participants spoke of a need for belonging; some offered the sentiment that as human beings we are relational and have a need to connect with others. Many of the Key Informants spoke about experiencing Métis pride, as well as pride in being a sexual or gender minority. A few younger participants spoke of feeling pride in their Métis heritage, though some youth were out of touch with Métis teachings and traditions. Some older participants discussed having more clear ties to Métis customs, with the Elder describing traditional culture as a “way of life,” and naming the importance of speaking Michif (a Métis language).

Participants drew from their knowledge of Métis and Indigenous histories to make sense of their gender and sexual identities also. Some participants named a cultural pride related to their queer status, feeling that contemporary mainstream culture had something to learn from Indigenous cultures’ progressive teachings around acceptance of sexual and gender diversity. These positive identity teachings also helped participants experience a sense of inclusion, feeling as though their Métis culture has room for them . . . as long as their community sticks to its roots.

### Resilience and healing

Though some noted that sigma and discrimination are waning over time, participants spoke of the need to engage in ongoing healing and self-care in the face of ongoing social harms. Acts of resilience included seeking out meaningful relationship and connection, sharing painful experiences with a non-judgmental listener, seeking allyship from family, friends, Elders and community members, and promoting positive education on sexual and gender diversity, perhaps drawing from Indigenous teachings. Connecting spiritually with creation through ceremony, Indigenous teachings, and time spent outdoors, were also named as wellness-promoting activities. Individuals also shared acts of care for self-such as exercising, taking a bath, watching comedy, and engaging in mental exercises such as setting boundaries—“Who cares what other people think!” (Youth, 16 years old) and positive self-talk—“Just be yourself” (Youth, 16 years old).

Importantly, two youth shared that learning to heal from anti-Indigenous racism and stigma helped them learn to heal from homophobia or transphobia. These youth noted that learning about challenges their Métis ancestors faced over the generations helped encourage them to be strong in the face of adversity. The Elder in this study offered the example of wearing the Métis Pride Sash publicly as a visual identifier of his queer identity within the Métis community, and as a sign of pride for his dual cultural minority identities—a unique emblem among Métis.

## Conclusion

The findings presented here echo those of other studies looking at promoting Indigenous mental health and addressing stigma. Other studies note that positive identity models, social connection, and cultural generativity—the sharing between generations—all combine to inform anti-stigma practice (Oturu, 2011; Snowshoe et al., 2015; Yang et al., 2007). Studies further note that cultural continuity between generations closes gaps created by colonial disruptions in culture cycles (Fryberg et al., 2018) and peer support for youth offers experiences of validation, safety, and authenticity to soothe social stressors related to stigma (Vandewalle et al., 2018). Some of these process-related elements are considered in a forthcoming paper on the methodologies used in this project, and reflect the theme of community research-as-healing. Again, drawing from the work of Hunt and Holmes (2015), engaging in decolonizing acts such as reconnecting with Indigenous ways of knowing, promoting solidarity across family and community members, and strengthening relationships between marginalized communities—Indigenous and queer in this case—offers pathways toward healing and resisting inherited colonial norms.

Shining Mountains and other similar community organizations can consider combatting shame through engaging the community in educational initiatives, such as through community circles, webinars, or podcasts, to inform the public about identity, stigma, and mental health constructs. They can offer mental health programs including multi-generational group circles for community members experiencing the stigma and discrimination described here, in order to facilitate strong community bonds, to combat the isolating effects of stigma, and to offer non-judgmental spaces for listening and sharing to promote pro-2SLGBTQ+ norms and attitudes (Deese & Dawson, 2013). The Métis Pride sash specifically can be used as a teaching tool as well as symbolically in community and leadership events, in order to signify allyship with members of the Métis queer community. Community gatherings can offer traditional teachings on acceptance of diversity to further bolster participants’ sense of identity and belonging and Indigenous pride. Policy at the agency and elsewhere can ensure the inclusion of anti-2SLGBTQ+ discrimination policies and ensure gender-inclusive language and action.
